# Self-reported perceived stress, depression, and generalized anxiety disorder among Kathak dancers and physically active non-dancers of North India

**DOI:** 10.3389/fpsyg.2023.1114377

**Published:** 2023-04-27

**Authors:** Monika Kulshreshtha, Kallur Nava Saraswathy, Nandita Babu, Shivani Chandel

**Affiliations:** ^1^Department of Sports Anthropometry, Sports Authority of India, Netaji Subhash Regional Centre, Lucknow, India; ^2^Department of Anthropology, University of Delhi, Delhi, India; ^3^Department of Psychology, University of Delhi, Delhi, India

**Keywords:** anxiety, dance therapy, depression, Kathak, stress, India

## Abstract

The aim of the present paper was to study the various common mental disorders in a sample of Kathak dancers and non-dancers of North India. 206 female Kathak dancers and 235 healthy controls, aged 18–45 years completed questionnaires assessing perceived stress (PSS-10), depressive symptoms (PHQ-9), and generalized anxiety (GAD-7). Pearson correlations assessed the association between perceived stress, depression, generalized anxiety, age, and years of dancing, and binary logistic regression identified the risk of developing depression and generalized anxiety disorder in Kathak dancers and non-dancers. The prevalence of perceived stress was similar among Kathak dancers and non-dancers. Kathak dancers reported significantly lower depressive symptoms compared to controls. Non-dancers with elevated perceived stress levels were 4 times more likely to report depressive symptoms and 7 times more likely to report anxiety symptoms, relative to dancers. The adjusted odds of reporting depressive symptoms along with generalized anxiety were higher among non-dancers compared to dancers. Kathak can be developed into a very effective psychotherapeutic tool for mitigating the risk of developing depression and generalized anxiety disorder.

## Introduction

Mental disorders are the psychological attributes that are reflected in an individual’s behavior and affect the normal development of a person’s culture (Celine and Antony, 2014; Poucher et al., 2021). Depression is one of the most common and serious psychiatric disorders which is associated with significant disability worldwide among people of all ages (Castro et al., 2015; World Health Organization, 2020). It not only affects the physical health, cognitive ability, behavior, and quality of life of an individual but at its worst, it can lead to suicide as well (Shumye et al., 2019). The global prevalence of the depressive disorder is around 3.2–4.7% and it is estimated that approximately 23 million adults would need care for depression at any given time in India (Moussavi et al., 2007; Arvind et al., 2019). Previously it has been observed that depression and generalized anxiety disorder (GAD) following the comorbidity rule are usually found together (Kessler et al., 1994; Wittchen et al., 2002; Cohen et al., 2014; Jensen et al., 2018; Kalin, 2020). Both depression and GAD are caused by several factors, chronic stress is one of the major reasons among both working and non-working individuals (Dantzer et al., 2008; Poucher et al., 2021). Excessive stress leading to the development of anxiety disorders precedes the occurrence of major depressive disorders (Kessler and Wang, 2008). The global prevalence of GAD is estimated to be 3.6% (World Health Organization, 2017) and is more common among females than males (4.6% compared to 2.6% at the global level; World Health Organization, 2017).

Depression and anxiety disorders are usually treated through first-line treatment methods like pharmacotherapy (National Collaborating Centre for Mental Health UK, 2010; Castro et al., 2015). In recent times, a combination of psychotherapy and pharmacotherapy is also used in primary care settings (Cuijpers, 2014). In addition to pharmacotherapy, recreational, cost-effective, and non-invasive psychotherapeutic treatment methods can also play a substantial role in preventing and treating mental health issues (e.g., depression, and anxiety). Dance is a form of exercise that is recreational and cost-effective as well. Previous studies have reported that dance as a form of exercise may significantly reduce the level of stress, anxiety, and depression and may improve the level of cognition by enhancing mood, confidence, and energy (Akandere and Demir, 2011; Kimura and Hozumi, 2012; Shinde et al., 2016; Laird et al., 2021; Salihu et al., 2021). It is a form of psychotherapy that uses creative movements to further the emotional, physical, cognitive, and social integration of the individual [American Dance Therapy Association (ADTA), 2020] and is based on the idea that both mind and body are correlational in nature (Quin et al., 2007). In India, there are a large number of classical dance forms like Odissi, Bharatnatyam, Kathakali, Kuchipudi, Manipuri, Mohiniyattam, and Kathak which have their own training regime, are deeply rooted in Indian tradition, focus on spirituality, act as a form of meditation (Chatterjee, 2013; Ministry of Culture, Government of India, 2020) and yet there is a dearth of even preliminary studies on the mental health of dancers in India (Shinde et al., 2016). Kathak is one such classical dance form that is prevalent in Northern India. It is a dynamic dance form in terms of dance therapy as all the body movements including footwork are therapeutic in nature. The fast footwork in Kathak acts as a medium to release anger and tension (Chatterjee, 2013). Kathak dance is also deeply rooted in spirituality and acts as a form of meditation (Ramaswamy and Deslauriers, 2014). All these qualities may place this dance form at an edge over other dance forms if it is used as psychotherapy.

Against this backdrop, the present paper aimed to study the common mental disorders (perceived stress, depression, and GAD) in a sample of Kathak dancers and physically active non-dancers of North India. Based on previous literature, we hypothesized that perceived stress, depression, and GAD would be less prevalent among Kathak dancers than non-dancers, and there would be less risk of developing given mental disorders among Kathak dancers than non-dancers.

## Materials and methods

### Study design

A cross-sectional study was conducted among 441 adult women aged 18–45 years from Delhi-NCR in North India. Data was collected using a purposive sampling technique.

### Participants

Participants included Kathak dancers and physically active non-dancers who were recruited from various Kathak dance institutes and universities. The mean age of Kathak dancers and non-dancers was 22.4 years. Kathak dancers included in this study had practiced this dance for 3 years, practiced >1 h daily, and had North Indian ancestry. Any dancer who was receiving training in any other form of dance had non-North Indian ancestry, had a severe physical chronic disease, and was currently pregnant and/or postpartum women were excluded (Kulshreshtha et al., 2021). Age, caste, and gender-matched physically active North Indians who exercised for >1 h daily (activities like brisk walking, jogging, cycling, running, gym, flexibility exercises, core exercises, yoga, cardio and involved in sports like badminton and volleyball, etc.) were recruited as non-dancers. Any non-dancers who were receiving training in any form of dance, had a sedentary lifestyle, non-North Indian ancestry, suffered from severe physical chronic disease, and were pregnant and/or postpartum women were excluded. After the screening, the final sample consisted of *n* = 206 Kathak dancers and *n* = 235 controls (Kulshreshtha et al., 2021). The study was approved by the Ethical Committee of the Department of Anthropology at the University of Delhi, India. The study was conducted according to the Helsinki guidelines for human subject research. Informed written consent was obtained from all participants prior to the collection of data.

### Measures

Data on age, time spent in respective activities, and years of learning dance were collected. For maintaining uniformity in data collection, all the questions in the following three questionnaires were asked by the same investigator individually to each of the participants after explaining the motive of that questionnaire:

The Perceived Stress Scale (PSS-10; Cohen et al., 1994) was used to assess the perception of stress among the sample. This instrument consisted of 10 items with a 5-point Likert scale. The total score ranges from 0 to 40. Higher scores corresponded to higher perceived stress as there were no cut-offs to determine stressed individuals and only comparisons can be done between participants. In the present study, the level of stress was analyzed based on quartiles of perceived stress: (i) No stress or less stress represented by the first quartile (0–13 for dancers; 0–16 for non-dancers), (ii) mild stress represented by second quartile (14–17 for dancers; 17–20 for non-dancers), (iii) moderate stress represented by third quartile (18–22 for dancers; 21–25 for non-dancers), and (iv) severe stress represented by fourth quartile (23–40 for dancers; 26–40 for non-dancers). The first and second quartiles were used as reference groups for analysis (Tavolacci et al., 2013). Cronbach’s α was calculated to assess internal consistency. Cronbach’s α for PSS-10 was 0.76 and 0.79 for Kathak dancers and controls, respectively.

The Patient Health Questionnaire (PHQ-9; Kroenke et al., 2001) was used to screen depression among participants. Its total score ranges from 0 to 27. Participants were categorized based on their total score: i) no depressive symptoms (0–4), ii) mild depressive symptoms (5–9), iii) moderate depressive symptoms (10–14), iv) moderately severe depressive symptoms (15–19), and v) severe depressive symptoms (20–27). Cronbach’s α for the PHQ-9 was 0.80 for Kathak dancers and 0.81 for non-dancers.

The GAD questionnaire (GAD-7; Spitzer et al., 2006) was used to screen for generalized anxiety among participants. This instrument consisted of 7 items measuring worry and anxiety symptoms with a 3-point Likert scale. Its total score ranges from 0 to 21. Higher scores corresponded to severe anxiety symptoms. Participants were categorized based on their total score: (i) no or minimal anxiety symptoms (0–4), (ii) mild anxiety symptoms (5–9), (iii) moderate anxiety symptoms (10–14), and (iv) severe anxiety symptoms (15–21). Cronbach’s α for the GAD-7 was 0.84 for Kathak dancers and 0.86 for non-dancers.

### Data analyses

Microsoft Excel version, 2010 and SPSS statistical package version 20 was used to conduct statistical analyses. All data passed the Kolmogorov–Smirnov test prior to analysis and were normally distributed. Basic descriptive statistics (mean and standard deviations) were calculated to assess the distribution of perceived stress, GAD, and depression across the sample. An independent sample *t*-test was used to examine the mean differences in PSS-10, PHQ-9, and GAD-7 scores between Kathak dancers and non-dancers. The chi-square test compared the categorical frequencies. Pearson’s correlation coefficients were calculated to determine the relationship between the PSS-10, PHQ-9, GAD-7, age, and years of learning dance. Results at *p* < 0.05 were considered significant. The effect size was calculated for each statistical test using the appropriate measures. A binary logistic regression analysis was conducted to determine the predictive ability of perceived stress for depression and GAD; GAD for depression.

## Results

### Distribution of perceived stress, depression, and GAD

The mean values of PSS-10 scores, PHQ-9 scores, and GAD-7 scores were in normal ranges but significantly lower in dancers compared to physically active non-dancers (*M* = 17.2 vs. 19.9, *p* < 0.001; *M* = 7.7 vs. 9.5, *p* < 0.001; *M* = 7.1 vs. 7.6, *p* = 0.241, respectively) (Table 1). There were no significant differences in the prevalence of perceived stress and GAD among Kathak dancers and non-dancers (46.1% vs. 46.8%, *p* = 0.92, 27.1% vs. 32.3%, *p* = 0.68). On the other hand, Kathak dancers reported significantly lower depressive symptoms compared to controls (23.4% vs. 42.9%, *p* < 0.001). A greater proportion of non-dancers reported severe and moderate depressive symptoms.

**Table 1:** Descriptive statistics, the prevalence of perceived stress, depression, and generalized anxiety disorder among Kathak dancers and Non-dancers of India.

Variables	Reference range (Normal values)	Kathak dancers (*N*=206)				Non-dancers (*N*=235)				*t (439)*	*p*	Hedges’ *g*
		*M*	*SD*	*n*	%	*M*	*SD*	*n*	%			
Age (years)	–	22.43	4.86		–	22.46	4.40		–	–0.08	0.934	0.01
Time spent in respective activity (hours/days)	–	2.50/7	–	1/7	–	1.93/7	–	1/7	–	–	–	–
Years of learning dance		6.41	1.50	–	–	–	–	–	–	–	–	–
PSS-10	KD < 18; ND < 21	17.2	6.6			19.9	6.5			−4.22	<.001^*^	0.41
PHQ-9	< 10	7.7	5.2			9.5	5.6			−3.53	<.001^*^	0.33
GAD 7	< 10	7.1	4.8	–	–	7.6	5.1	–	–	−1.17	0.241	–
PSS-10 Categories			*χ*^2^ *(3)*	*p*	Cramer’s *v*
No stress	KD 0-13; ND 0-16	–		54	26.2	–		66	28.1	0.45	0.928	–
Mild stress	KD 14-17; ND 17-20	–		57	27.7	–		59	25.1			
Moderate stress	KD 18-22; ND 21-25	–		55	26.7	–		65	27.7			
Severe stress	KD 23-40; ND 26-40	–		40	19.4	–		45	19.1			
PHQ-9 Categories										*χ*^2^ *(4)*	*p*	Cramer’s *v*
No depressive symptoms	0–4	–		63	30.6	–		40	17.0	23.30	<0.001^*^	0.23
Mild depressive symptoms	5–9	–		95	46.1	–		94	40.0			
Moderate depressive symptoms	10–14	–		24	11.7	–		59	25.1			
Moderately severe depressive symptoms	15–19	–		16	7.8	–		25	10.6			
Severe depressive symptoms	20–27	–		8	3.9	–		17	7.2			
GAD-7 Categories										*χ*^2^ *(3)*	*p*	Cramer’s *v*
No anxiety	0–4	–		79	38.2	–		81	34.5				1.488	0.689	–
Mild anxiety	5–9	–		71	34.3	–		78	33.2			
Moderate anxiety	10–14	–		38	18.4	–		52	22.1			
Severe anxiety	15–21	–		18	8.7	–		24	10.2			

PSS-10 = Perceived stress scale; PHQ-9 = Patient Health Questionnaire; GAD-7 = Generalized Anxiety Disorder, KD = Kathak dancer; ND = Non-dancer. *Significant at *p* < 0.05.

The distribution of participants with depressive and non-depressive symptoms; anxiety and non-anxiety symptoms according to their perceived stress level were assessed (Table 2). 30.5% of Kathak dancers and 62.7% of non-dancers having either moderate or severe levels of stress possessed depressive symptoms, indicating that non-dancers were at high risk for depression.

**Table 2:** Distribution of participants with depressive and no-depressive symptoms; anxiety and no-anxiety symptoms according to their perceived stress level.

Variables	Kathak dancers						Non-dancers					
	Stress		No- stress		*χ*^2^ *(1)*	*p*	Stress		No- stress		*χ*^2^ *(1)*	*p*
	*n*	*%*	*n*	*%*			*n*	*%*	*n*	*%*		
Depressive symptoms	29	30.52	19	17.11	5.15	0.23^*^	69	62.72	32	25.6	32.91	< 0.001^*^
No depressive symptoms	66	69.47	92	82.88			41	37.27	93	74.4		
Total	95	100	111	100			110	100	125	100		
Anxiety	25	44.6	70	46.66	–	0.876	59	77.63	51	32.07	–	< 0.001^*^
No anxiety	31	55.35	80	53.33			17	22.36	108	67.92		
Total	56	100	150	100			76	100	159			

^*^Significant at *p* < 0.05.

Further 44.6% of Kathak dancers and 77.6% of non-dancers having either moderate or severe levels of stress possessed generalized anxiety symptoms, indicating that non-dancers were at high risk for GAD as well (Table 2).

### Correlation and binary logistic regression

PHQ-9 scores and GAD-7 scores were significantly positively correlated with PSS-10 scores among both dancers and non-dancers (*r* = 0.37, *p* < 0.001; *r* = 0.60, *p* < 0.001; *r* = 0.40, *p* < 0.001; *r* = 0.43, *p* < 0.001, respectively). GAD-7 scores were significantly positively correlated with PHQ-9 scores among both dancers and non-dancers (*r* = 0.61, *p* < 0.001; *r* = 0.70, *p* < 0.001, respectively) (Table 3). In dancers, PHQ-9 scores, GAD-7 scores, and PSS-10 scores were significantly negatively correlated with years of learning dance and age. In non-dancers, there was no significant correlation of age with PSS-10, PHQ-9, and GAD-7.

**Table 3:** Pearson correlation coefficients between PHQ-9, GAD-7, and PSS-10 among Kathak dancers and non-dancers.

Mental health variables	Pearson correlation coefficients (*r*)			
	*PHQ-9*		*GAD-7*	
	Kathak dancers	Non-dancers	Kathak dancers	Non-dancers
PSS-10	0.374^**^	0.604^**^	0.400^**^	0.430^**^
GAD-7	0.611^**^	0.709^**^	–	–

PSS-10 = Perceived stress scale; PHQ-9 = Patient Health Questionnaire; GAD-7 = Generalized Anxiety Disorder. ^**^ Significant at *p* < 0.001.

Binary logistic regression analysis revealed that with elevated stress, the adjusted odds of being at risk for developing depression were higher in non-dancers compared to dancers (OR = 4.89; 95% CI [2.80, 8.54]; *p* < 0.001 vs. OR = 2.11; 95% CI [1.08, 4.13]; *p* = 0.028). Further, both unadjusted and adjusted odds of being at risk for developing GAD were higher with perceived stress, in non-dancers (OR = 7.06; 95% CI [3.70, 13.48]; *p* < 0.001 vs. OR = 4.44; 95% CI [2.079, 9.498]; *p* < 0.001). The odds of developing depression due to GAD was also higher in non-dancers compared to dancers (OR = 12.09; 95% CI [6.19, 23.61]; *p* < 0.001 vs. OR = 4.44; 95% CI [2.07, 9.49]; *p* < 0.001, respectively) (Table 4).

**Table 4:** Unadjusted and adjusted odds ratio (95% confidence interval) of PHQ-9 with PSS-10, GAD-7 with PSS-10 and PHQ-9 among Kathak dancers and non-dancers.

Mental health variables	Models	PHQ-9			GAD-7		
		Exp (B) (OR)	Significance	95% C. I	Exp (B) (OR)	Significance	95% C. I		
		Kathak dancers					
PSS-10	Model 1	2.128	0.025*	1.100–4.113	0.922	0.795	0.497–1.708
	Model 2	2.117	0.028*	1.084–4.135	0.954	0.888	0.494–1.841
PHQ-9	Model 1	–	–	–	3.485	< 0.001*	1.758–6.906
	Model 2	–	–	–	4.444	< 0.001*	2.079–9.498
		Non-dancers					
PSS-10	Model 1	5.278	< 0.001*	2.953–9.431	7.349	< 0.001*	3.899–13.855
	Model 2	4.891	< 0.001*	2.801–8.541	7.068	< 0.001*	3.705–13.485
PHQ-9	Model 1	–	–	–	12.098	< 0.001*	6.198–23.616
	Model 2	–	–	–	12.098	< 0.001*	6.198–23.616

Model 1 = Unadjusted OR; Model 2 = Adjusted OR; Adjusted for age, occupation, education, and smoking status in Kathak dancers and education in non-dancers; OR = Odds Ratio; PSS-10 = Perceived stress scale; PHQ-9 = Patient Health Questionnaire; GAD-7 = Generalized Anxiety Disorder.

*Significant at value of *p* < 0.05.

## Discussion

The present paper studied the common mental disorders (perceived stress, depression, and GAD) in a sample of Kathak dancers and physically active non-dancers of North India. At the beginning of the study, it was hypothesized that perceived stress, depression, and GAD would be less prevalent among Kathak dancers than physically active non-dancers, and there would be less risk of developing given mental disorders among Kathak dancers than non-dancers.

Findings revealed that the mean scores of PSS-10 were found to be in the normal ranges and lower than the reported mean scores in previous studies conducted among dancers and non-dancers (Shinde et al., 2016; Pangtey et al., 2020). For perceived stress, in contrast to our hypothesis, Kathak dancers reported similarly elevated rates of stress to those reported for non-dancers and dancers of other dance styles (Krasnow, 1999; Tomei et al., 2015; Zulaikha et al., 2018). It has been reported previously that when dance is taken up as an occupation, the life of a dancer becomes highly stressful and challenging (Micheli et al., 1984; Hamilton et al., 1989; Hanna, 2017). As the opportunities for taking up classical dancing as a career have tremendously increased in India, it has led to an increase in competition (Financial Express, 2019) which in turn has given rise to the notion of having an ideal body shape (Karathanou et al., 2021) and may have made dancers more susceptible to perceived stress. Thus, in Kathak dancers, the physical, psychological, and esthetic demands (e.g., long working hours, low financial rewards; body shape concerns) imposed on them may have acted as stressors due to increased competition (Chatterjee et al., 2015; Kulshreshtha et al., 2021). Further, in the present study, the high level of perceived stress among Kathak dancers may be due to the positive stress (eustress) which motivates them to perform better (Franco, 2018) and thrive in a dance industry fraught with competition; however, more future research in this area is warranted.

Compared with physically active controls, dancers reported lower rates of depressive symptoms despite having similarly high levels of perceived stress as non-dancers. In support of our hypothesis, the odds of developing depressive symptoms due to high perceived stress were lower among dancers compared to non-dancers. Further, the level of both stress and depression decreased with an increase in age and years of dancing. These results suggest that Kathak being a spiritual form of dance (Ramaswamy and Deslauriers, 2014), was playing an important role in managing stress and preventing the risk of developing depression. Similar results depicting a reduction in the level of stress and depression via practicing dance have been reported extensively in previous studies (Akandere and Demir, 2011; Pinniger et al., 2012; Hanna, 2017; Karkou et al., 2019; Karathanou et al., 2021).

In terms of anxiety, in the current study, GAD was assessed among Kathak dancers and non-dancers. The proportion of individuals having moderate and severe GAD was less among Kathak dancers than non-dancers. However, there was no significant difference in the mean scores of GAD-7 among dancers and non-dancers. The prevalence of anxiety among Kathak dancers may be attributed to performance anxiety. The findings of the present study also showed that GAD decreases with an increase in years of learning dance. In the present study, GAD was found to have a high correlation with perceived stress and depression, and this result was consistent with previous studies conducted among other dance forms (Liu et al., 2017). Further, in support of our hypothesis, the odds of developing GAD due to high perceived stress were lower in Kathak dancers. This reduction in the risk of the development of generalized anxiety would have further reduced the chances of the development of depression in individuals. As previous studies have reported that the chances of depression increase in individuals suffering from anxiety disorder (Kalin, 2020). In the present study as well the risk of developing depressive symptoms was found to be higher among those suffering from GAD; though this risk was considerably lower among Kathak dancers.

Thus, findings from the current study provide additional evidence that (a) Kathak dancers are less susceptible to depression and GAD, relative to physically active controls in females. (b) Age and years of dance training may influence the extent of depression and GAD in dancers. (c) Kathak dancers are less susceptible to the development of depression when suffering from GAD, thus making the condition less lethal.

To the best of our knowledge, this was the first study to examine depression, GAD, and perceived stress among any type of Indian dance form in general and Kathak in particular. However, this study has certain limitations. First, the data collected was self-reported, based on three well-established questionnaires. These are screening tools and should be followed by interviews in the clinical assessment of depression, generalized anxiety, and perceived stress. Secondly, the current study did not take stressors and types of stress (eustress/distress) into account. Future research should aim to focus on stressors as well as conduct clinical interviews to understand the underlying causes of perceived stress, depression, and GAD. Thirdly, though Kathak dancing is practiced by both men and women, the present study could be conducted only among women. Fourthly, sample collection was not based on the phenotype but based on dancers and non-dancers which might have created some sample biases but still, it hints toward the association between the three studied variables of mental health and dance and the authors recommend replicating such study in a larger sample size.

Despite high stress which might be occupational, Kathak was still playing an important role in mitigating the risk of developing depression and GAD among dancers. Thus, it can be developed into a very effective psychotherapeutic tool that will not only be non-invasive but cost-effective in reducing the risk of developing depression and GAD in non-dancers. Kathak dance focuses on oneness with God and has various therapeutic movements in its core structure like tapping of feet, and coordinated hand and body movements. This attribute of Kathak can be used in the form of therapeutic exercise or coping methods by all age groups and will yield the best results if started very early in life. Utilizing the results of the present study, future research can further study the role of the Kathak dance form as a psychotherapeutic tool of intervention among individuals having depression or GAD which will further establish its utility as one of the efficient coping methods in combating depression and GAD.

## Data availability statement

The original contributions presented in the study are included in the article/supplementary material, further inquiries can be directed to the corresponding author/s.

## Ethics statement

The studies involving human participants were reviewed and approved by Department of Anthropology, University of Delhi, Delhi, India. The patients/participants provided their written informed consent to participate in this study.

## Author contributions

MK has made a substantial contribution in the acquisition, analysis, or interpretation of data for the article, drafted the article, and revised it. MK and SC have made a substantial contribution to the concept or design of the study. SC, KS, and NB critically evaluated the manuscript for important intellectual content and provided feedback. All authors contributed to the article and approved the submitted version.

## Funding

This work was supported by the University Grants Commission (UGC) of India by providing financial assistance for collection of data.

## Conflict of interest

The authors declare that the research was conducted in the absence of any commercial or financial relationships that could be construed as a potential conflict of interest.

## Publisher’s note

All claims expressed in this article are solely those of the authors and do not necessarily represent those of their affiliated organizations, or those of the publisher, the editors and the reviewers. Any product that may be evaluated in this article, or claim that may be made by its manufacturer, is not guaranteed or endorsed by the publisher.
